# Cell‐to‐cell communication: microRNAs as hormones

**DOI:** 10.1002/1878-0261.12144

**Published:** 2017-10-26

**Authors:** Recep Bayraktar, Katrien Van Roosbroeck, George A. Calin

**Affiliations:** ^1^ Department of Experimental Therapeutics The University of Texas MD Anderson Cancer Center Houston TX USA; ^2^ Center for RNA Interference and Non‐Coding RNAs The University of Texas MD Anderson Cancer Center Houston TX USA; ^3^ Department of Leukemia The University of Texas MD Anderson Cancer Center Houston TX USA

**Keywords:** cell–cell communication, circulating miRNAs, exosomes, extracellular vesicles, microRNAs, tumor microenvironment

## Abstract

Mammalian cells can release different types of extracellular vesicles (EVs), including exosomes, microvesicles, and apoptotic bodies. Accumulating evidence suggests that EVs play a role in cell‐to‐cell communication within the tumor microenvironment. EVs’ components, such as proteins, noncoding RNAs [microRNAs (miRNAs), and long noncoding RNAs (lncRNAs)], messenger RNAs (mRNAs), DNA, and lipids, can mediate paracrine signaling in the tumor microenvironment. Recently, miRNAs encapsulated in secreted EVs have been identified in the extracellular space. Mature miRNAs that participate in intercellular communication are released from most cells, often within EVs, and disseminate through the extracellular fluid to reach remote target cells, including tumor cells, whose phenotypes they can influence by regulating mRNA and protein expression either as tumor suppressors or as oncogenes, depending on their targets. In this review, we discuss the roles of miRNAs in intercellular communication, the biological function of extracellular miRNAs, and their potential applications for diagnosis and therapeutics. We will give examples of miRNAs that behave as hormones.

**Abbreviations**
CAAs: cancer‐associated adipocytes
CAFs: cancer‐associated fibroblasts
CLL: chronic lymphocytic leukemia
ECM: extracellular matrix
EVs: extracellular vesicles
FGF2: fibroblast growth factor 2
HDL: high‐density lipoproteins
LDL: low‐density lipoproteins
miRNAs: microRNAs
MVs: microvesicles
NFs: normal fibroblasts
RISC: RNA‐induced silencing complex
TAMs: tumor‐associated macrophages
TGF‐β: transforming growth factor‐β
TLDA: TaqMan Low‐Density Arrays
TLRs: toll‐like receptors
TME: tumor microenvironment
TMVs: tumor‐derived MVs
VEGF: vascular endothelial growth factor

## Introduction

MicroRNAs (miRNAs) are a class of short noncoding RNAs that play key roles in almost all biological pathways in mammalian and other organisms (Ambros, 2004; Bartel, 2004). miRNAs are 19–25 nucleotides in length and can regulate gene expression both at the transcriptional and at translational level by repressing target genes (Ambros, 2004; Bartel, 2004). Initially, miRNAs are transcribed as thousand‐base‐long primary transcripts by RNA polymerase II and are called precursor miRNAs (Ambros, 2004; Bartel, 2004). Precursor miRNAs are exported to the cytoplasm via exportin 5, where they are integrated into DICER and RNA‐induced silencing complex (RISC), which includes argonaute proteins. Finally, mature miRNA strands specifically bind to partially complementary sequence motifs in the 3′ untranslated region of target mRNA (Ambros, 2004; Bartel, 2004). In 2002, we demonstrated that a coding gene on a region of the long arm of chromosome 13, at 13q14, was frequently deleted in chronic lymphocytic leukemia (CLL) and that this deletion caused the loss of two miRNAs (miR‐15a and miR‐16‐1) (Bullrich *et al*., 2001). Subsequently, we reported that the chromosome 13q14 region was deleted in more than 65% of CLL patients and that allelic loss in this region correlates with downregulation of both miR‐15 and miR‐16 expression (Calin *et al*., 2002). This finding opened new opportunities for noncoding RNAs in cancer research.

Extracellular vesicles (EVs) are small membrane vesicles, such as ectosomes, microparticles, microvesicles (MVs), tumor‐derived MVs (TMVs), exosomes, and oncosomes that are produced by different mechanisms and can be released from almost all cell types (Cocucci and Meldolesi, 2011; Raposo and Stoorvogel, 2013; Redis *et al*., 2012; Valadi *et al*., 2007). EVs participate in cell–cell communication and can typically be classified based on their size (from 4 to 10 microns), intracellular origin, and density (Cocucci and Meldolesi, 2011; Raposo and Stoorvogel, 2013; Valadi *et al*., 2007). EVs can be found in all different body fluids, such as blood, serum, plasma, saliva, urine, and pleural effusions (Fernandez‐Mercado *et al*., 2015; Liu *et al*., 2017a; Mitchell *et al*., 2008; Ortiz‐Quintero, 2016; Weber *et al*., 2010). In the last decade, several studies have shown that EVs are enriched for various proteins, such as cytokines, messenger RNAs, lipids, and noncoding RNAs, such as miRNAs and long noncoding RNAs (lncRNAs) (Colombo *et al*., 2013; Valadi *et al*., 2007). The content of EVs, which are shed by almost all cell types under both physiological and pathological conditions, can be transported from a parental cell to neighboring cells (Arita *et al*., 2008; Cocucci and Meldolesi, 2011; Thery *et al*., 2009; Trajkovic *et al*., 2008). Through their protein, RNA, and DNA cargoes, EVs can regulate important biological functions of recipient cells, such as proliferation, angiogenesis, and apoptosis, processes that are deregulated in human cancers (Clayton *et al*., 2004; Kanlikilicer *et al*., 2016; Rashed *et al*., 2017; Valadi *et al*., 2007).

In this review, we summarize the current knowledge regarding the contributions of miRNAs in secreted EVs, their potential clinical and therapeutic applications, biological significance, relationship with tumors, and roles in cell–cell communication.

## How cells communicate

**Figure 1:**
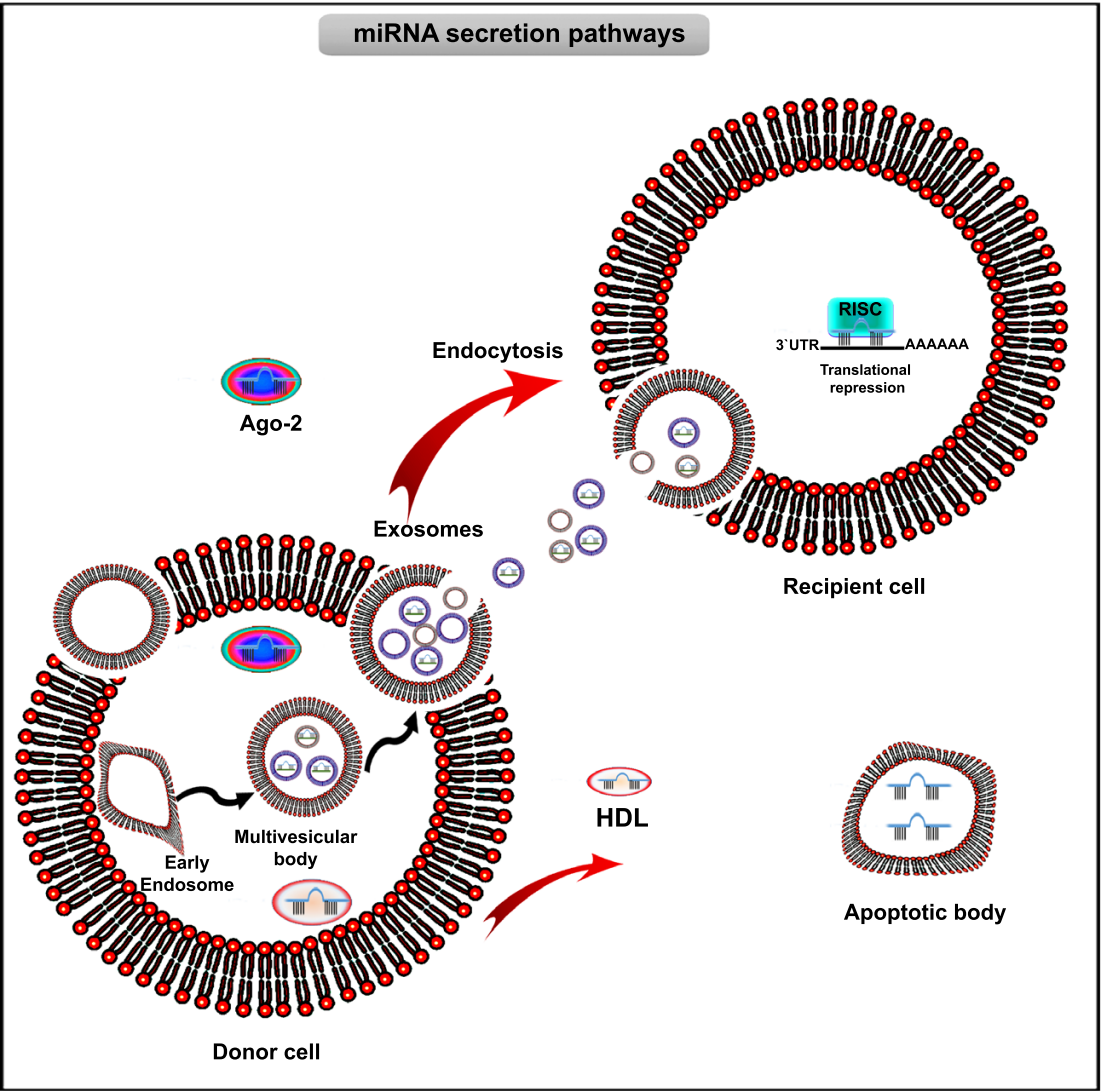
MicroRNAs release and uptake mechanism between donor and recipient cells. Biogenesis of exosomes. Early endosomes originate from the cell membrane via endocytosis. Multivesicular bodies originate by invagination of the plasma membrane. Multivesicular bodies fuse with the plasma membrane and exosomes are released into the extracellular space. Some types of miRNAs are generally localized in membrane‐derived vesicles (exosomes, microvesicles, apoptotic bodies), while some miRNAs are found mainly in miRNA‐binding protein complexes, such as Ago‐2, or high‐density lipoproteins (HDL). Finally, miRNAs enter into recipient cells and interact with specific target genes.

The best‐known mechanisms of cell–cell communication are chemical receptor‐mediated events, direct cell–cell interaction, and cell–cell synapses (Valadi *et al*., 2007). In recent years, an additional mechanism has been identified via extracellular vesicles (Fig. 1), which will be described in the following sections.

### EVs: an alternative type of cell–cell communication

Exosomes are the best characterized class of EVs; they are secreted by almost all cell types, are 40 to 100 nm in diameter, and are enriched in a 1.13–1.19 g·mL^−1^ fraction in a sucrose density gradient (Kanada *et al*., 2015; Turturici *et al*., 2014). The protein and lipid composition of exosomes reflects their cellular sources (Staubach *et al*., 2009; Yim *et al*., 2016). The most common exosomal proteins are annexins, tetraspanins (CD63, CD81, CD82, and CD9), and heat‐shock proteins (Hsp60, Hsp70, and Hsp90). In 2007, Valadi *et al*. showed a new mechanism of cell–cell communication: delivery of RNA by transfer through exosomes. They demonstrated that exosomes carry both mRNAs and miRNAs when released from mouse and human mast cells and that many of these RNAs seem to be packaged exclusively into exosomes. Zhang *et al*. (2010) have been reported that cells can selectively package miRNAs into MVs and secreted miRNAs can act as signaling molecules mediating intercellular communication. In their study, it has been demonstrated that miR‐150 (a leukocyte and lymphocyte‐specific miRNA) was selectively packaged into MVs and exosomal miR‐150 entered the recipient cells (Zhang *et al*., 2010). Exosomal miR‐150 derived from THP1 cells can enter and deliver into human microvascular endothelial HMEC‐1 cells, and delivered miR‐150 dramatically reduced c‐Myb expression level and promoted cell migration (Zhang *et al*., 2010).

### High‐density lipoproteins as carriers of extracellular miRNAs

Circulating miRNAs can be transported from donor cells to recipient cells by exosomes, microvesicles, apoptotic bodies, lipoproteins, and ribonucleoproteins (such as nucleophosmin 1 and argonaute 2) (Boon and Vickers, 2013; Vickers and Remaley, 2012). New studies have revealed that high‐density lipoproteins (HDL) and low‐density lipoproteins (LDL) can transport miRNAs into cells as well (Vickers *et al*., 2011). In 2011, Vickers *et al*. demonstrated that several types of RNAs, such as miRNAs and tRNAs, could be detected in HDL and LDL particles. They profiled approximately 700 miRNAs in pure human HDL particles using real‐time PCR‐based TaqMan Low‐Density Arrays (TLDA) (Vickers *et al*., 2011) and found that miR‐135a*, miR‐188‐5p, and miR‐877 were upregulated in healthy control HDL particles, while miR‐223, miR‐105, and miR‐106a were upregulated in HDL particles from patients with familial hypercholesterolemia. In addition, they showed that HDL‐mediated miRNA‐223 transport represses cholesterol uptake by direct binding to the 3′‐UTR of the scavenger receptor BI (*SR‐BI*) mRNA and controls its expression and function (Vickers *et al*., 2011). Tabet *et al*. (2014) demonstrated that miR‐223 is transferred from native HDL to endothelial cells and HDL inhibits expression of intercellular adhesion molecule 1 (ICAM‐1) through the transfer of miR‐223 to endothelial cells. All these findings support that HDL‐miRNA delivery is a new mechanism of miRNA transport.

## Transfer of biological information between tumor microenvironment and malignant cells via miRNAs

It has long been known that small molecules, cytokines, and growth factors mediate functional interactions between cancer cells and the tumor microenvironment (Schoepp *et al*., 2017). However, recent studies have revealed that cancer cells also transfer functional information at the paracrine level using EVs. In fact, it appears that the cargo of EVs can influence the stroma by activating molecular pathways, which are at least partially different from those modulated by soluble factors. The tumor microenvironment (TME) plays critical roles in the initiation, development, and progression of cancer. The TME includes extracellular matrix as well as different types of cells, including cancer‐associated fibroblasts (CAFs), tumor‐associated macrophages (TAMs), endothelial cells, pericytes, and immune cells (Berindan‐Neagoe and Calin, 2014; Friedl and Alexander, 2011; Laberge *et al*., 2012).

### miRNAs and cancer‐associated fibroblasts

Fibroblasts are the most common constituent in human tissues and tumors, and they regulate tissue repair and inflammation during wound healing (Kalluri and Zeisberg, 2006). Previous studies have reported that activated tumor fibroblasts or cancer‐associated fibroblasts are phenotypically and functionally different from normal fibroblasts (Zhang *et al*., 2017). CAFs have been shown to stimulate cancer progression and proliferation through helping create the extracellular matrix (ECM) and through the secretion of a variety of cytokines, chemokines, and growth factors, such as vascular endothelial growth factor (VEGF), transforming growth factor‐β (TGF‐β), and fibroblast growth factor 2 (FGF2) (Kalluri, 2016; Kalluri and Zeisberg, 2006; Orimo *et al*., 2005; Shen *et al*., 2016). Accumulating evidence suggests that miRNAs play key roles in the activation and transition of fibroblasts (Aprelikova *et al*., 2013; Mitra *et al*., 2012).

Recent studies have shown deregulated miRNA expression in CAFs from clinical specimens. For instance, in 2012, Mitra *et al*. demonstrated that miR‐155 was upregulated, while miR‐31 and miR‐214 were downregulated, in ovarian CAFs when compared with primary human normal omental fibroblasts or primary human CAFs. They found that triple transfection of these miRNAs with mimics activated tumor‐promoting functions, such as migration, invasion, and colony formation, in primary human normal omental fibroblasts (Mitra *et al*., 2012). In 2014, Yeung *et al*. used next‐generation sequencing to show that miR‐21 expression is upregulated in exosomes and tissue isolated from cancer‐associated adipocytes (CAAs) and CAFs (Au Yeung *et al*., 2016). Moreover, it has been shown that exosomes can be secreted by CAFs and taken up by cancer cells, as isolated exosomes from primary CAFs transfected with labeled miR‐21 successfully entered ovarian cancer cells (Au Yeung *et al*., 2016). They confirmed transfer of miR‐21 from primary CAFs to ovarian cancer cells by co‐culturing CAFs transfected with labeled miR‐21 with ovarian cancer cells (Au Yeung *et al*., 2016). In addition, miR‐21 was frequently upregulated in CAFs from both human pancreatic ductal adenocarcinoma samples and primary cell cultures, and higher expression of miR‐21 was significantly correlated with decreased overall survival in pancreatic ductal adenocarcinoma patients (Donahue *et al*., 2014; Kadera *et al*., 2013). In esophageal squamous cell carcinoma, miR‐21 expression in tumor tissues was found to be primarily localized in the stroma adjacent to the cancer cells (Nouraee *et al*., 2013). We further showed that miR‐21 could be secreted by fibroblasts in the microenvironment and taken up by cancer cells, resulting in increased migration and invasion potential of esophageal tumor cells (Nouraee *et al*., 2013). However, the exact mechanism of miR‐21 secretion and transportation was not explored. Finally, miR‐21 was found to induce the expression of CAF markers, suggesting its involvement in the ‘activation’ of fibroblasts to CAFs (Nouraee *et al*., 2013).

**Figure 2:**
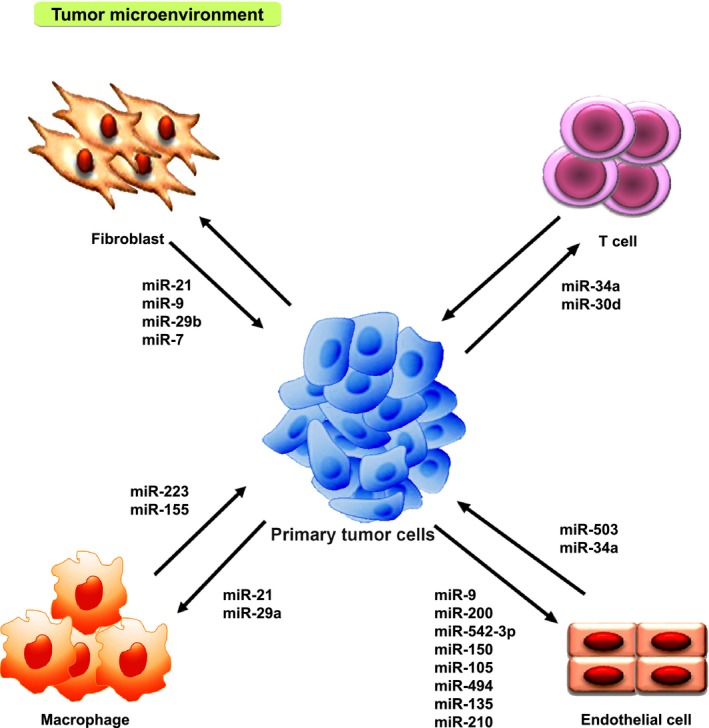
Schematic illustration of the interaction between primary tumor cells and tumor microenvironment through miRNAs. miRNAs can play a key role in cell–cell communication in several physiological and pathophysiological processes associated with many human diseases, including cancer. Selected examples of paracrine miRNA signaling between primary tumor cells, immune cells, and endothelial cells are shown.

Yang *et al*. (2014) investigated the miRNA signatures of CAFs and normal fibroblasts isolated from human gastric cancer tissue, and they reported that miR‐34b, miR‐93, miR‐301a, and miR‐106b were significantly upregulated, while miR‐483‐3p, miR‐26a, let‐7g, miR‐148a, miR‐145, miR‐424, and miR‐214 were significantly downregulated in CAFs compared with corresponding normal fibroblasts. To understand the clinical significance of miR‐106b expression, they analyzed a subset of patients with gastric cancer and found that overall survival was dramatically higher in patients with low miR‐106b expression than in patients with high miR‐106b expression (Yang *et al*., 2014). Baroni *et al*. (2016) revealed that in patients with different breast cancer subtypes (luminal‐A, luminal‐B, HER2, and triple negative), miR‐9 was upregulated in primary triple‐negative breast CAFs when compared to normal fibroblasts (NFs). They also demonstrated that miR‐9 secreted from tumors can be transferred to recipient normal fibroblasts via exosomes and that miR‐9 uptake leads to increased cell motility in normal fibroblast cells that overexpress this miRNA (Baroni *et al*., 2016). They co‐injected MDA‐MB‐468 triple‐negative breast cancer cells with either normal fibroblasts or normal fibroblasts that overexpressed miR‐9 into the mammary fat pad of 8‐week‐old female SCID mice, and found that tumor volume was significantly increased in mice co‐injected with MDA‐MB‐468 breast cancer cells and normal fibroblasts overexpressing miR‐9, compared with the control group (Baroni *et al*., 2016). These findings showed that higher expression levels of miR‐9 in fibroblasts affect breast cancer progression and provide new insights into the role of miR‐9 in the crosstalk between cancer cells and stroma (Baroni *et al*., 2016). (Donnarumma *et al*. (2017) showed that the release of specific miRNAs from CAF exosomes can promote oncogenic signaling in breast cancer. Their results demonstrated that three miRNAs (miR‐21, miR‐143, and miR‐378e) were significantly upregulated in CAF exosomes in respect to NF exosomes (Donnarumma *et al*., 2017). When T47D luminal‐A breast cancer cells were treated with exosomes isolated from NFs and CAFs and labeled with PKH26, they found that T47D cells are able to take up exosomes derived from CAFs and NFs (Donnarumma *et al*., 2017). Furthermore, they observed that CAF exosomes treatment significantly promoted stemness, EMT, invasiveness capacity, and anchorage‐independent cell growth (Donnarumma *et al*., 2017). Pang *et al*. (2015) showed that miR‐155 was upregulated in exosomes derived from pancreatic cancer cells. They co‐cultured primary mouse pancreatic fibroblast cells with BxPC‐3 and SW1990 pancreatic cancer cells and showed that normal fibroblasts could convert into CAFs after miR‐155 had been taken up (Pang *et al*., 2015). They demonstrated that miR‐155 targets the TP53INP1 protein, leading to increased alpha‐SMA protein expression in normal fibroblasts (Pang *et al*., 2015). Altogether, their results indicated that miR‐155 in exosomes secreted from pancreatic cancer cells might activate normal fibroblasts by targeting TP53INP1 (Pang *et al*., 2015). In conclusion, CAFs regulate tumorigenesis in many cancer types through exosome‐mediated delivery of specific miRNAs (Fig. 2).

### miRNAs and tumor‐associated macrophages

Macrophages originate from precursor blood monocytes and play key roles in the immune system. Depending on their polarization state, they can perform distinct functions in different biological processes. Traditionally, macrophages can be categorized as either classically activated (M1) macrophages or alternatively activated (M2) macrophages (Biswas and Mantovani, 2010; Lawrence and Natoli, 2011; Mosser and Edwards, 2008). M1 macrophages play key roles in the pro‐inflammatory and antitumor responses and are induced by toll‐like receptors (TLRs), IFN‐γ, and lipopolysaccharide (LPS), while M2 macrophages are activated by IL‐4 and IL‐13 and are responsible for angiogenesis, tissue remodeling, and tumor progression (Mantovani *et al*., 2007; Zhang *et al*., 2012).

Tumor‐associated macrophages (TAMs) are generally composed of M2‐polarized macrophages and associated with poor clinical outcomes in patient with various cancer types (Noy and Pollard, 2014; Steidl *et al*., 2010). TAMs share many similarities with the M2‐polarized macrophages population and can affect different protumoral functions, such as promotion of angiogenesis, matrix remodeling, secretion of growth factors, and suppression of inflammation and adaptive immunity (Binnemars‐Postma *et al*., 2017; Sica *et al*., 2006). Recent studies showed that new molecular players, miRNAs, have an important role in the polarization of TAMs and on monocyte/macrophage lineage development (Baer *et al*., 2016; Li *et al*., 2015; Wang *et al*., 2015). (Baer *et al*. (2016) showed that deletion of the miRNA‐processing enzyme DICER regulates TAM programming. They generated Dicer1‐knockout mice and employed orthotropic mammary carcinoma, subcutaneous lung and colorectal carcinoma models. Macrophage‐specific Dicer1 deletion significantly delayed tumor progression. They demonstrate that let‐7‐5p family members were upregulated in Dicer‐knockout and wild‐type TAMs (Baer *et al*., 2016). These findings suggest that Dicer1 deletion can regulate the antitumor phenotype of M2 TAMs to M1‐like TAMs in different types of tumor models (Baer *et al*., 2016). Squadrito *et al*. (2012) described that the human and mouse mannose receptor *MRC1* gene encodes miR‐511‐3p, an intronic miRNA, and showed that miR‐511‐3p and *MRC1* are transcriptionally co‐regulated. They overexpressed miR‐511‐3p in BM‐derived hematopoietic cells and injected Lewis lung carcinoma (LLC) cells in mice and were able to show that overexpression of miR‐511‐3p inhibited tumor growth in the LLC model (Squadrito *et al*., 2012). (Sonda *et al*. (2013) identified 79 differentially expressed miRNAs in either tumor‐infiltrating versus normal CD11b^+^ cells purified from the spleen of healthy mice. They found that miR‐142‐3p and miR‐150 were the most downregulated miRNAs in tumor‐infiltrating CD11b^+^ cells and computationally determined genes that are involved in monocyte–macrophage commitment, as well as potential target genes of miR‐142‐3p (Sonda *et al*., 2013). They further showed that miR‐142‐3p directly regulates gp130, which is a common subunit of the interleukin‐6 cytokine receptor family that regulates macrophage generation (Sonda *et al*., 2013). A comprehensive list of extracellular miRNAs shuttling between cancer cells and their neighboring cells can be found in Table 1.

**Table 1:** MicroRNAs as hormones: extracellular miRNAs shuttling between cancer cells and their neighboring cells

miRNA	Donor cells	Accepting cells	Target/Pathway	Function/impact	References
miR‐21,‐143,‐378	CAFs	Breast cancer cells	EMT	Promotes the stemness and EMT	Donnarumma *et al*. (2017)
miR‐21	CAFs	Ovarian cancer cells	APAF1	Stimulates cancer cell motility and invasion	Au Yeung *et al*. (2016)
miR‐146a	CAFs	Pancreatic cells	Snail	Increases proliferation	Richards *et al*. (2017)
miR‐9	CAFs	Normal fibroblasts	CDH1	Enhances cell motility	Baroni *et al*. (2016)
miR‐29b	CAFs	Breast cancer cells	CCL11 and CXCL14	Inhibits breast cancer cellular viability and metastasis	Liu *et al*. (2017b)
miR‐409	CAFs	Prostate cancer cells	RSU1 and STAG2	Induces cell growth and EMT	Josson *et al*. (2015)
miR‐133b	CAFs	Normal fibroblasts	IL6 and TGF‐β	Promotes fibroblast activation	Doldi *et al*. (2015)
miR‐320a	CAFs	Hepatocellular carcinoma cells	PBX3	Inhibits tumor progression	Zhang *et al*. (2017)
miR‐221	CAFs	Breast cancer cells	IL6/Stat3	Promotes hormonal therapy resistance	Sansone *et al*. (2017)
miR‐124	CAFs	Oral cancer cells	CCL2 and IL‐8	Promotes cell growth and migration	Li *et al*. (2017)
miR‐7	CAFs	Head and neck cancer cells	RASSF2	Enhances cell proliferation and migration	Shen *et al*. (2017)
miR‐101	CAFs	Hepatocellular cancer cells	TGF‐βR1, Smad2, and VE‐cadherin	Inhibits vascular mimicry formation	Yang *et al*. (2016)
miR‐141,‐146b‐5p	CAFs	Breast cancer cells	p16	Represses the migration and invasiveness	Al‐Khalaf and Aboussekhra (2017)
miR‐1, ‐206, ‐31	CAFs	Lung cancer cells	FOXO3a/VEGFA/CCL2	Promotes migration and tumor growth	Shen *et al*. (2016)
miR‐214	CAFs	Ovarian cancer cells	CCL5	Activate tumor‐promoting functions	Mitra *et al*. (2012)
miR‐940	TAMs	Ovarian cancer cells	CD206 and CD163	Tumor‐promoting function	Chen *et al*. (2017)
miR‐21	TAMs	Gastric cancer cells	PTEN	Suppresses cell apoptosis and enhances activation of PI3K/AKT	Zheng *et al*. (2017)
miR‐720	TAMs	Breast cancer cells	GATA3	M2 polarization	Zhong and Yi (2016)
miR‐155	TAMs	Hepatocellular cancer cells	C/EBPbeta	Suppresses cytokine production	He *et al*. (2009)
miR‐511‐3p	TAMs	Macrophage expressing MRC1 cells	ROCK2	Inhibits tumor growth and alters tumor blood vessel morphology	Squadrito *et al*. (2012)
let‐7b	TAMs	Prostate cancer TAMs	IL‐12, IL‐23, IL‐10 TNF‐α, and CXCL12	Inhibits macrophage migration and tumor growth	Li *et al*. (2015)
miR‐125a	TAMs	Epithelial cells	FIH1 and IRF4	Enhances M1 and attenuates M2 polarization	Zhao *et al*. (2016)

## miRNAs function as toll‐like receptors ligands

Toll‐like receptors (TLRs) are highly conserved pathogen recognition receptors that play a key role in the active innate immune system of all multicellular organisms (Takeda *et al*., 2003). TLRs are widely expressed in different cell types, including macrophages, dendritic cells (DCs), neutrophils, natural killer cells, and fibroblasts (Batool *et al*., 2016; Visintin *et al*., 2001). TLRs recognize conserved structural motifs of pathogens (pathogen‐associated molecular patterns, PAMPs) including flagellins, nucleic acids, lipoproteins, saccharides, and peptidoglycans (Akira, 2009). In addition, TLRs induce chronic inflammation and regulate the maintenance of tissue homeostasis. Despite their structural and functional similarities, each member of the TLR family recognizes specific ligands and has a different cellular localization. TLR1, 2, 4, 5, 6, and 10 are expressed on cell surfaces that are involved in the recognition of cell surface molecules, while TLR3, 7, 8, and 9 are located intracellularly and recognize nucleic acids.

Previous studies showed that TLRs, especially TLR7 and TLR8, recognize GU‐rich single‐stranded RNA (ssRNA) derived from human immunodeficiency virus‐1 (HIV‐1) in certain cell types, such as dendritic cells and B lymphocytes, which leads to cell activation and IFN‐α release (Heil *et al*., 2004; Lund *et al*., 2004). In 2012, Fabbri *et al*. showed that tumor‐secreted miRNAs are involved in intercellular communication in the tumor microenvironment as they can bind and activate TLR7 and TLR8 (Fabbri *et al*., 2012). They found that miR‐16, miR‐21, miR‐27b, and miR‐29a were highly expressed in exosomes (Fabbri *et al*., 2012). Interestingly, miR‐16 was upregulated in exosomes from derived HEK cells, while miR‐21 and miR‐29a were upregulated in exosomes from derived non‐small‐cell lung cancer cells (Fabbri *et al*., 2012). They observed that lung cancer‐specific miR‐21 and miR‐29a bind to TLR7 and TLR8 and activate TLR‐mediated NF‐κB signaling, as well as increase secretion of the prometastatic and pro‐inflammatory cytokines TNF‐α and IL‐6 (Fabbri *et al*., 2012). Lehmann and colleagues showed that extracellular let‐7 is a potential activator of TLR7 signaling in both immune cells and neurons (Lehmann *et al*., 2012). They found that extracellular let‐7 interacts with TLR7, which is expressed on central nervous system (CNS) neurons, and they detected *TLR7* mRNA in the mouse brain cortex (Lehmann *et al*., 2012). These findings suggest that in addition to cancer‐specific exosomal miRNAs, let‐7 can bind to TLR7 and plays a key role in neurodegenerative diseases such as Alzheimer disease.

## Circulating miRNAs as biomarkers

Several types of malignancies require early and sensitive detection in order to ensure effective treatment and management of the patients. Abnormal expression of specific miRNAs was observed during the progression of several types of cancer (Fabbri *et al*., 2008). Accumulating evidence demonstrates that specific miRNAs hold great promise as novel biomarkers for clinical diagnosis of many types of diseases, including cancer (Berindan‐Neagoe *et al*., 2014). Especially, circulating miRNAs are remarkably stable in the blood. Therefore, they can be extracted from blood and measured in blood or other bodily fluids for each patient (Cheng, 2015; Larrea *et al*., 2016). To measure circulating miRNAs, methods such as qRT‐PCR (LNA‐based, TaqMan, or other proprietary technologies), digital PCR (dPCR), microarrays, and next‐generation sequencing (NGS) are used (Larrea *et al*., 2016; Van Roosbroeck *et al*., 2013).

### Hematological cancers

As circulating miRNAs represent a class of ideal biomarkers for hematological cancer diagnosis, many studies have been performed to determine their potential and are reviewed elsewhere (Chen *et al*., 2014a; Lawrie, 2013). In this section, we give a few examples of specific circulating miRNAs that have been shown to be altered in the plasma of hematological cancer patients. The oncogenic role of especially miR‐21, miR‐155, miR‐150, and miR‐210 are well established in hematological malignancies (Fabbri *et al*., 2008; Fernandez‐Mercado *et al*., 2015; Munker and Calin, 2011). In 2008, Lawrie *et al*. compared miR‐155, miR‐21, and miR‐210 expression levels in serum samples from diffuse large B‐cell lymphoma (DLBCL) patients with those of healthy controls, and found that circulating miR‐155, miR‐21, and miR‐210 levels were significantly upregulated in DLBCL patients and that serum levels of miR‐21 are associated with relapse‐free survival in DLBCL. Additionally, levels of miR‐21 were upregulated in DLBCL cell lines and clinical specimens of DLBCL (Chen *et al*., 2014b). These findings suggest that miR‐21 might function as a biomarker for the diagnosis of DLBCL. Our previous studies showed that miR‐155 was significantly overexpressed in monoclonal B‐cell lymphocytosis (MBL) when compared with normal B cells and that miR‐155 overexpression in plasma is a predictor of poor response to therapy (Ferrajoli *et al*., 2013). Similar studies have reported that miR‐150 and miR‐342 were significantly downregulated in the plasma of acute myeloid leukemia (AML) patients at diagnosis compared to healthy controls and that these microRNAs are candidate biomarkers and potential predictors of relapse in AML (Fayyad‐Kazan *et al*., 2013). In addition, circulating miR‐192 has been found to be significantly downregulated in patients with chronic lymphocytic leukemia (CLL) compared with healthy individuals (Fathullahzadeh *et al*., 2016). Finally, Filip *et al*. (2017) showed that miR‐34a‐5p, miR31‐5p, miR‐155‐5p, miR‐150‐5p, miR‐15a‐3p, and miR‐29a‐3p were upregulated in serum samples of CLL patients compared with healthy individuals.

### Solid cancers

Several studies using microarray platforms to investigate miRNA biomarker potential in solid tumors were published, among them the study of Volinia and coworkers, who described a large‐scale detailed analysis of the miRNA profiles in 540 samples from six solid tumors: breast, colon, lung, pancreas, prostate, and stomach (Volinia *et al*., 2006). This screening showed that miR‐21, miR‐191, and miR‐17‐5p are significantly upregulated in all of the tumor types and that miR‐29b‐2, miR‐223, miR‐128b, miR‐199a‐1, miR‐24‐1, miR‐24‐2, miR‐146, miR‐155, miR‐181b‐1, miR‐20a, miR‐107, miR‐32, miR‐92, miR‐214, miR‐30c, miR‐25, miR‐221, and miR‐106a were overexpressed in three or more types of solid cancers (Volinia *et al*., 2006). After identifying the presence of miRNAs in solid tumors, circulating miRNA signatures have been widely described in many cancer types (Chen *et al*., 2008; Lawrie *et al*., 2008; Mitchell *et al*., 2008). For instance, Hannafon *et al*. showed that miR‐1246 was upregulated in exosomes from breast cancer cells compared to normal mammary epithelial cells and mouse plasma. When they analyzed human plasma samples, miR‐1246 and miR‐21 were detected at significantly higher levels in the plasma exosomes of 16 breast cancer patients as compared to the plasma exosomes of healthy control samples (Hannafon *et al*., 2016). In another example, Zhang *et al*. (2015) identified 66 circulating miRNAs that were upregulated, while 32 circulating miRNAs were downregulated in serum samples of ovarian cancer patients compared with serum samples from healthy controls. More specifically, miR‐497, miR‐16‐2*, miR‐195, and miR‐2861 were expressed at significantly higher levels in serum samples of ovarian cancer patients compared to cervical intraepithelial neoplasia patients and healthy control subjects (Zhang *et al*., 2015). Xu *et al*. (2016) identified 13 miRNAs deregulated in plasma in patients with PDAC: miR‐106b‐3p, miR‐126‐3p, miR‐1271, miR‐1285, miR‐19b‐3p, miR‐26b‐3p, miR‐296‐5p, miR‐486‐5p, miR‐663B, miR‐7–5p, miR‐938, miR‐942, and miR‐181c‐5p. Of particular interest was miR‐486‐5p, which showed diagnostic value in pancreatic cancer patients when compared with healthy controls and patients with chronic pancreatitis (Xu *et al*., 2016). In addition, Zhang *et al*. (2016) report that miR‐1246 and miR‐1290 were overexpressed in serum samples of patients with non‐small‐cell lung cancer (NSCLC) when compared to healthy individuals. After overexpression of these miRNAs in lung epithelial cells, ectopic overexpression of miR‐1246 repressed *PRL36A*,* GLIPR1*,* HAS2*,* NCKAP5*,* MT1G,* and *CYP4F11*, whereas overexpression of miR‐1290 repressed *MT1G*,* MT1H*,* GLIPR1*,* CYP4F11,* and *NCKAP5* (Zhang *et al*., 2016). Furthermore, they identified the putative tumor suppressor *MT1G* as a direct target of miR‐1246 and miR‐1290 [76]. Their observations indicate that miR‐1246 and miR‐1290 can behave as noninvasive biomarkers that may be used for the early detection of lung cancer (Zhang *et al*., 2016). Finally, Zhu *et al*. (2016) showed that the serum levels of miR‐182, miR‐183, and miR‐210 were significantly upregulated and that miR‐126 levels were significantly downregulated in NSCLC patients compared with healthy controls.

## Novel miRNA‐based therapeutic approaches

As cancer‐specific miRNAs are master regulators of many critical oncogenic pathways, their utilization for cancer diagnosis and as promising potential therapeutic target for clinical treatment strategies or personalized medicine is becoming more and more clear (Ling *et al*., 2013). After discovering the role of miRNAs in cell–cell signaling and TME, targeting miRNAs as anticancer therapeutic strategy is becoming more realistic and promising. Several groups designed miRNA overexpression or inhibition systems as a therapeutic strategy, and this approach led to promising outcomes *in vivo* (Bayraktar *et al*., 2017; Hydbring *et al*., 2017; Mangala *et al*., 2016). For instance, Mangala *et al*. showed that miR‐106b‐5p, miR‐30c‐5p, and miR‐141‐3p were significantly upregulated in ovarian cancer‐associated endothelial cells compared to normal endothelial cells. Silencing of these miRNAs leads to restoration of tight junction function and in turn decreases angiogenesis. To evaluate the *in vivo* biological effects of miR‐106b‐5p and miR‐30c‐5p silencing, they established an ovarian cancer orthotopic mouse model and found that miR‐106b‐5p inhibitor treatment resulted in 50% reduction in tumor growth, while miR‐30c‐5p inhibitor treatment resulted in ~25% reduction in tumor growth in ovarian cancer orthotopic mouse model (Mangala *et al*., 2016). The combination of miRNA mimic or inhibitor delivery with chemotherapeutic drugs has recently been used to enhance the efficiency of treatment in several types of tumor. Recently, we showed that overexpression of miR‐155 induces chemoresistance in lung cancer cells and acute lymphoblastic leukemia cells (Van Roosbroeck *et al*., 2017). Additionally, miR‐155‐overexpressing tumors became resistant to cisplatin treatment in an orthotopic lung cancer mouse model (Van Roosbroeck *et al*., 2017). Administration of miR‐155 inhibitor alone significantly reduced number of tumors, tumor size, and aggregate mass of metastasis, but when we combined miR‐155 inhibitor with cisplatin treatment, the chemotherapy resistance was almost completely reversed (Van Roosbroeck *et al*., 2017).

Another novel approach is the combination of small interfering RNA (siRNA) and miRNA‐based treatment that can allow a ‘boosting’ effect for targeting oncogenic pathways and repressing cancer growth (Nishimura *et al*., 2013). We proved that combined inhibition of EphA2, an ovarian cancer oncogene, with EphA2 siRNA and miR‐520d‐3p mimic exhibits synergistic effects (Nishimura *et al*., 2013). We identified miR‐520d‐3p as a tumor suppressor upstream of EphA2, which is associated with longer overall and relapse‐free survival time in ovarian cancer patients (Nishimura *et al*., 2013). In this study, miR‐520d‐3p was shown to directly target EphA2 and EphB2 and inhibit their protein expression in ovarian cancer cells. Treatment with the combination of miR‐520d‐3p mimic and EphA2 siRNA resulted in less invasion/migration by HeyA8 and SKOV3ip1 cells than either miR‐520d‐3p or EphA2 siRNA alone or the controls. Moreover, *in vivo* therapeutic delivery of miR‐520d‐3p mimic and EphA2 siRNA induced potent synergy, resulting in substantial inhibition of tumor growth when compared with individual treatments in ovarian cancer tumor xenograft models (Nishimura *et al*., 2013).

Currently, some miRNA‐associated therapies are being evaluated in ongoing Phase I clinical trials in cancer (Van Roosbroeck and Calin, 2017). The first miRNA‐based cancer therapy was a liposome‐formulated synthetic miR‐34a mimic (MRX34) (ClinicalTrials.gov, ID: NCT01829971). Because of multiple immune‐related severe adverse events observed in cancer patients receiving MRX34, this study has been terminated early (Van Roosbroeck and Calin, 2017). miRNAs can target multiple genes simultaneously and lead to unexpected side effects and unwanted toxicities. However, combination therapies of miRNAs with siRNA and/or chemotherapy can reduce the risks of adverse events and increase therapeutic synergy as compared with monotherapy.

## Conclusion

Since the discovery of the first miRNA, several thousands of miRNAs have been identified in humans, and studies on miRNAs have increased, remarkably during the last decade. Furthermore, miRNAs are frequently deregulated in human diseases including cancer, which offers many opportunities for diagnosis, prognosis, and treatment of human diseases. Recently, it was found that miRNAs are released by donor cells, play a key role in the process of cell‐to‐cell communication, influence the phenotype of recipient cells, and likely reach many distant tissues. Taken together, these miRNAs can serve as valuable biomarkers for various pathological conditions.
